# Normalization of the Lymph Node T Cell Stromal Microenvironment in lpr/lpr Mice Is Associated with SU5416-Induced Reduction in Autoantibodies

**DOI:** 10.1371/journal.pone.0032828

**Published:** 2012-03-06

**Authors:** Susan Chyou, Sha Tian, Eric H. Ekland, Theresa T. Lu

**Affiliations:** 1 Autoimmunity and Inflammation Program, Hospital for Special Surgery, New York, New York, United States of America; 2 Pediatric Rheumatology, Hospital for Special Surgery, New York, New York, United States of America; 3 Department of Microbiology and Immunology, Weill Medical College of Cornell University, New York, New York, United States of America; Beth Israel Deaconess Medical Center, United States of America

## Abstract

The vascular-stromal elements of lymph nodes can play important roles in regulating the activities of the lymphocytes within. During model immune responses, the vascular-stromal compartment has been shown to undergo proliferative expansion and functional alterations. The state of the vascular-stromal compartment and the potential importance of this compartment in a spontaneous, chronic model of autoimmunity have not been well studied. Here, we characterize the vascular expansion in MRL-lpr/lpr lymph nodes and attempt to ask whether inhibiting this expansion can interfere with autoantibody generation. We show that characteristics of vascular expansion in enlarging MRL-lpr/lpr lymph nodes resemble that of the VEGF-dependent expansion that occurs in wild-type mice after model immunization. Surprisingly, treatment with SU5416, an inhibitor of VEGF and other receptor tyrosine kinases, did not have sustained effects in inhibiting vascular growth, but attenuated the anti-dsDNA response and altered the phenotype of the double negative T cells that are expanded in these mice. In examining for anatomic correlates of these immunologic changes, we found that the double negative T cells are localized within ectopic follicles around a central B cell patch and that these T cell-rich areas lack the T zone stromal protein ER-TR7 as well as other elements of a normal T zone microenvironment. SU5416 treatment disrupted these follicles and normalized the association between T zone microenvironmental elements and T cell-rich areas. Recent studies have shown a regulatory role for T zone stromal elements. Thus, our findings of the association of anti-dsDNA responses, double negative T cell phenotype, and altered lymphocyte microenvironment suggest the possibility that lymphocyte localization in ectopic follicles protects them from regulation by T zone stromal elements and functions to maintain autoimmune responses. Potentially, altering the lymphocyte microenvironment that is set up by the vascular-stromal compartment can be a means by which to control undesired autoimmune responses.

## Introduction

Lymph nodes are sites of immune responses, and, within lymph nodes, the activities of and interactions among immune cells are supported and regulated by a highly plastic vascular-stromal compartment that can expand and undergo phenotypic alterations during immune responses. The mechanisms that regulate these vascular-stromal changes and how they contribute to the progression and regulation of the immune response are just beginning to be better understood ([1]–[4], [5], [6]). In lupus and other autoimmune diseases, lymph nodes can undergo hypertrophy. Abnormal tissue architecture or immune cell localization in lymphoid tissues from patients with or in mouse models of systemic autoimmune diseases have been described [7]–[15], but alterations and potential significance of the vascular-stromal compartment in these settings are not yet well understood.

The anatomic compartmentalization of B cells to polarized follicles at the cortex and T cells and dendritic cells to the “T zone” in the paracortex within lymph nodes is in part is dictated by the unique identity of fibroblastic reticular cells (FRCs) in each compartment. Specialized FRCs within the follicles express B cell-attracting CXCL13, while T zone FRCs express CCR7 ligands that promote the localization of CCR7-expressing T cells and dendritic cells to the T zone [1],[2],[3],[16],[17]. The FRCs of each compartment are specialized in other ways as well, with T zone FRCs capable of having a regulatory role and limiting T cell proliferation or activation [6], [18]–[23]. The T zone FRCs also express the extracellular matrix constituents and ensheathe a reticular network of collagen-rich fibrils [1], [2], [16]. One of the matrix proteins expressed by the T zone FRCs is a protein recognized by the antibody ER-TR7, which is also expressed highly in the plasma cell-rich medulla, but notably normally is excluded from the B cell follicles [24]–[26]. During model immune responses in wild-type mice, well-delineated ectopic follicles with distinct B and T cells zones have been described to appear in the medulla. ER-TR7 is expressed within the T cell areas, suggesting that the association between ER-TR7 and T zone areas remains intact in these immunization-induced ectopic follicles [27].

The blood vessels of lymph nodes bring in cells, nutrients, and oxygen. The high endothelial venules (HEVs) are specialized postcapillary venules that are the portals of entry for circulating lymphocytes and are mostly found in the T zone and medulla [28]. Upon acute immunization, HEVs and other portions of the blood vessels undergo a proliferative expansion that is dependent on vascular endothelial growth factor (VEGF) and mediated initially by CD11c+ cells and then by B and T cells together. The process is rapid, with the initial burst of proliferation occurring 2 days after immunization and significant expansion occurring by day 5 [29], [30]. Thereafter, there is a re-establishment of vascular quiescence, whereby the vasculature may continue to expand if the stimulus is of a chronic nature (such as antigen emulsified in CFA) but the rate of proliferation is attenuated. Along with the downregulation of proliferation is downregulation of VCAM-1 on HEV endothelial cells and, corresponding to this phenotypic alteration, the efficiency with which HEV allow lymphocytes to enter. This re-establishment of vascular quiescence is mediated by late-accumulating CD11c^hi^ presumed dendritic cells [31]. These and other vascular alterations have been studied mainly in the context of antigen/adjuvant and infection models [32]–[35]. Lymph node vascular growth and functional alterations in a spontaneous chronic lupus model has not been characterized, and the potential therapeutic value of inhibiting the growth and other alterations to autoantibody production is unknown.

In this paper, we characterize the vascular expansion that occurs with progressive lymph node enlargement in the MRL/lpr model of lupus and attempt to inhibit the vascular growth to ask about the subsequent effects on autoantibody generation. The MRL/lpr model is characterized by massive lymph node enlargement in part due to the accumulation of CD4-CD8-B220+ syndecan+ (herein referred to as “double negative”) T cells [9], [36], [37]. Surprisingly, treatment with the receptor tyrosine kinase inhibitor SU5416 that inhibits VEGF receptors as well as other receptor tyrosine kinases did not have sustained effects on inhibiting vascular growth and yet was able to attenuate the anti-dsDNA response and downregulate syndecan on the double negative T cells. We found that double negative T cells were localized around a central B cell area within ectopic follicles and that, unlike normal T cell-rich areas, the T cell area within these follicles seemed to exclude ER-TR7 and other T zone constituents. SU5416 treatment led to disruption of these follicles and normalized the association between T cells and T zone stromal and other constituents. Our results indicate an association between unique alterations of the vascular-stromal compartment and autoimmunity and suggest the possibility that manipulating the association of lymphocytes with the vascular-stromal compartment could be used to regulate undesired autoimmune responses.

## Materials and Methods

### Ethics statement

The Institutional Animal Use and Care Committee of the Hospital for Special Surgery specifically approved this study and all animal procedures were performed in accordance with their regulations.

### Mice

MRL+/+ and MRL-lpr/lpr mice were either obtained from The Jackson Laboratory (Bar Harbor, ME) or bred at HSS from Jackson Laboratory breeders. For the initial characterization of the vascular expansion and the 2 and 11.5 week SUGEN compound treatments, the JAX 006825 strain was used. This strain of MRL-lpr/lpr mice demonstrated an attenuated lymphoproliferative phenotype and longer life spans than had been typical for their MRL-lpr/lpr mice (see http://jaxmice.jax.org/strain/006825.html). The experiments utilizing 4–5 weeks of SU5416 treatment used JAX strain 000485, which were the rederived MRL-lpr/lpr line that had the more severe phenotype typical of the original MRL-lpr/lpr mice.

### SUGEN compound treatment

MRL-lpr/lpr mice were injected intraperitoneally with 500 ug per 50–100 ul of SU5416 or SU1498 (Sigma, St. Louis, MO) or DMSO vehicle every other day for indicated time periods.

### Flow cytometry analysis

For all of our flow cytometric analyses except analyses of plasma cells, we prepared single cell suspensions by digesting with collagenase type II (Worthington, Lakewood, NJ) and stained cells as described previously [30]. For proliferation studies, mice were given intraperitoneal injections of 2 mg of BrdU at 18 hours and 1 hour prior to sacrifice and 0.8 mg/ml BrdU in the drinking water in between injections as previously described [30], [31]. For analysis of plasma cells, single-cell suspensions were obtained by mashing lymph nodes through a 70 µm filter and staining with antibodies for flow cytometry. Cells were analyzed using a FACSCanto (BD Biosciences) and CellQuest Pro (BD Biosciences) software.

### Immunohistochemistry

Lymph nodes were flash-frozen and 7-µm sections were cut on a cryostat. Sections were dried for 1 h, fixed for 10 min in acetone and immunohistochemical staining of fresh-frozen sections was performed as described previously [30]. HRP or alkaline phosphatase-conjugated secondary Abs were from Jackson ImmunoResearch Laboratories.

### Antibodies

All antibodies used were from BD Biosciences (San Jose, CA) unless otherwise specified. Abs used were against CD45, CD31, peripheral node addressin (PNAd), gp38 (BioLegend, San Diego, CA, or Developmental Studies Hybridoma Bank, Iowa City, IA), CD11c (HL3), B220, CD19, IgD, CD21/CD35, CD3, syndecan, BrdU, ER-TR7 (BaChem, Bubendorf, Switzerland), and LYVE-1 (Angio-Proteomie Boston, MA). Biotinylated peanut agglutinin (PNA) was from Vector Laboratories (Burlingame, CA).

### VEGF measurements

For lymph node VEGF levels, popliteal lymph nodes were solubilized in modified RIPA buffer and subjected to VEGF measurement using a commercial kit (R&D Systems, DuoSet mouse VEGF) as previously described [30].

### Serum titer ELISA

Anti-dsDNA ELISA was as described [38]. Briefly, double-stranded DNA was prepared using the S1 nuclease method. Plates were pre-coated with poly-L-lysine and then dsDNA before addition of serum samples. For total IgG titers, plates were blocked with 1% BSA in PBS prior to addition of serum samples. Bound IgG was detected with anti-mouse IgG HRP (Jackson ImmunoResearch) and chromogenic substrate 3,3′,5,5′-Tetramethylbenzidine (Sigma-Aldrich, St. Louis, MO).

### Anti-dsDNA ELISPOT

Plates were coated with poly-L-lysine and dsDNA as for ELISAs before adding cells suspended in RPMI with 2% FCS (Invitrogen, Carlsbad, CA) for 4 hours. The rest of the assay was carried out as described [39], with detection of bound IgG/M by anti-mouse IgG/M-biotin (Jackson ImmunoResearch), streptavidin-alkaline phosphatase (Jackson ImmunoResearch), and chromogenic substrate 5-bromo-4-chloro-3-indolyl-phosphate (Sigma). Spots per well were counted and back-multiplied to total organ counts to generate spots per organ.

## Results

### Enlarged MRL-lpr/lpr lymph nodes demonstrate vascular expansion and state of re-established quiescence

**Figure 1:**
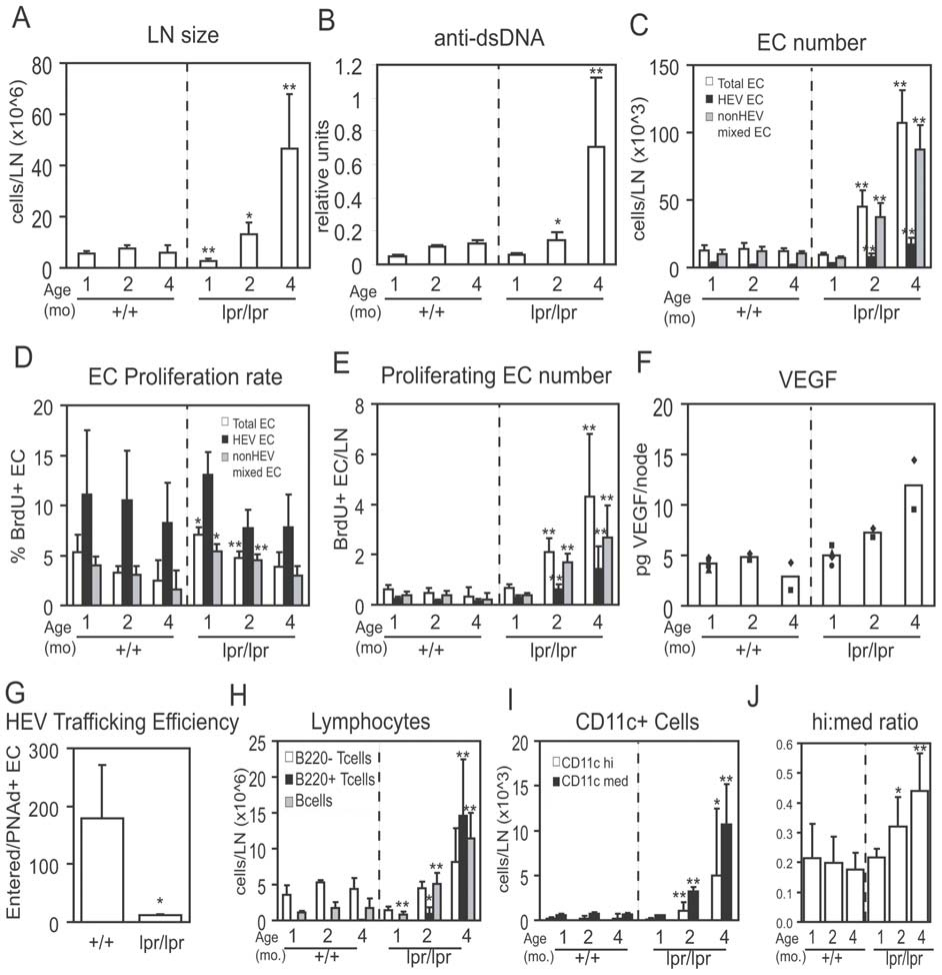
MRL-lpr/lpr lymph node vasculature shows expansion and re-established quiescence with course of disease. MRL+/+ and MRL-lpr/lpr mice were examined at indicated ages; brachial lymph nodes were used for flow cytometry-based studies (A, C–E, G–J) and popliteal lymph nodes were used for VEGF determination (F). (A) Lymph node cellularity as determined by count of lymph node cells. (B) Serum anti-dsDNA IgG titers. (C) Number of endothelial cells per lymph node as determined by flow cytometry. “Total EC” = CD45^neg^CD31^pos^ cells; total endothelial cells are comprised of PNAd ^pos^ “HEV EC” and PNAd ^neg^ “nonHEV mixed EC.” (D) Endothelial cell proliferation rate as determined by the percent of endothelial cells that are BrdU+. (E) Number of proliferating (BrdU+) endothelial cells per lymph node. (F) VEGF levels in lymph nodes. Each symbol represents 1 mouse. (G) HEV trafficking efficiency. About 3×10^7^ CFSE-labeled splenocytes were intravenously injected into 3.5 month old mice at 30 minutes prior to sacrifice. HEV trafficking efficiency was defined as the number of CFSE-labeled splenocytes that entered the lymph node divided by the number of HEV endothelial cells. n = 3 mice per condition. Representative of 3 similar experiments. (H) Number of lymphocyte subsets over time. Cells were identified as follows: “B220+ T cells” were B220^pos^CD3^pos^ cells; “B220− T cells” were B220^neg^CD3^pos^ cells; “B cells” were B220^pos^CD3^neg^ cells. (I) Number of subsets of CD11c+ cells over time. (J) Ratio of CD11c^hi^ cells to CD11c^med^ cells over time. For A–E, H–J, n = 6 mice per condition over 3 experiments. For A–J, * = p<.05 and ** = p<.01 in comparison to the same measurement in the age matched MRL+/+ mice using t-test.

To understand whether lymph node enlargement in lupus-prone MRL-LPR/LPR/lpr mice was accompanied by vascular growth and whether the characteristics of the vascular growth was similar to that after immunization, we used flow cytometry to examine endothelial cells over time, gating as we have done in other studies [29]–[31], [40] on CD45^neg^CD31^pos^ cells to delineate the total endothelial cell population (“Total EC”). Within this population, endothelial cells can be divided into peripheral node addressin (PNAd) ^pos^ and PNAd ^neg^ cells. PNAd marks HEV endothelial cells [28], [41], and thus we refer to PNAd ^pos^ endothelial cells here as “HEV endothelial cells”. PNAd ^neg^ endothelial cells represent a mixture of mostly blood endothelial cells and less lymphatic endothelial cells (about a 60∶40 mixture in brachial lymph nodes in wild-type mice [29]) and are termed “non-HEV mixed endothelial cells.” As lymph node cellularity (Fig. 1A) and anti-dsDNA titers (Fig. 1B) increased in MRL-lpr/lpr mice over time, endothelial cell numbers increased concomitantly (Fig. 1C). Both HEV and non-HEV mixed endothelial cells expanded in numbers over time (Fig. 1C).

We examined whether the increase in endothelial cell numbers involved an increase in proliferation. Mice were pulsed with BrdU for 18 hours and endothelial cell proliferation rate was determined by the percentage of endothelial cells that were BrdU ^pos^. Endothelial cell proliferation rates were modestly higher in MRL-lpr/lpr lymph nodes, reflecting mainly higher proliferation in the non-HEV mixed endothelial cell population (Fig. 1D). However, because endothelial cell numbers were expanding in the MRL-lpr/lpr mice, the number of proliferating endothelial cells actually increased as lymph nodes became enlarged in these mice (Fig. 1E). Lymph node vascular expansion in MRL-lpr/lpr mice, then, was accompanied by an increase in the number of proliferating endothelial cells, although the rate of proliferation was only modestly increased over that of MRL+/+ mice. VEGF levels were greater in the enlarged lymph nodes of MRL-lpr/lpr mice (Fig. 1F), suggesting that, similar to lymph nodes stimulated by model immunizations [30], [40], VEGF might contribute to the vascular proliferation and expansion.

Upon acute immunization, lymph node endothelial cells undergo a period of re-established quiescence after the initial proliferative burst and expansion [30], [31]. We entertained the possibility that we had missed any acute proliferative burst in MRL-lpr/lpr nodes because we had examined over long time intervals and that the slightly increased rate of endothelial cell proliferation observed in the MRL-lpr/lpr lymph nodes reflected a period of re-established vascular quiescence. We examined HEV trafficking efficiency and found that 15-fold fewer naïve lymphocytes were able to enter lymph nodes per HEV endothelial cell in MRL-lpr/lpr mice when compared to MRL-+/+ mice (Fig. 1G). Together, the modestly increased rate of endothelial cell proliferation and the reduced HEV trafficking efficiency were consistent with the idea that the lymph node vasculature in MRL-lpr/lpr mice at 2 and 4 months of age were in a period of re-established vascular quiescence.

We have shown that different CD11c^pos^ populations play distinct roles in regulating lymph node vascular growth and quiescence after immunization [29], [31]. CD11c^med^ cells play a predominant role in the initiation of vascular growth while CD11c^hi^ presumed dendritic cells are critical for the subsequent re-establishment of vascular quiescence and stabilization [29], [31]. At homeostasis, CD11c^hi^ cells are at low numbers relative to CD11c^med^ cells. Both populations accumulate over the course of a primary immune response, with CD11c^med^ cells initially outpacing the CD11c^hi^ cells during the initiation of vascular expansion. With the onset of vascular quiescence and stabilization, CD11c^hi^ cells accumulate more rapidly and the ratio of CD11c^hi^ cell: CD11c^med^ cell increases [31]. In the MRL-lpr/lpr nodes, lymphocytes, including the double negative T cells, accounted for the majority of the cells (Fig. 1H). However, both CD11c^hi^ and CD11c^med^ cells also accumulated (Fig. 1I), and the ratios of CD11c^hi^ cell: CD11c^med^ cell were increased at 2 and 4 months (Fig. 1I–J). Together, these results suggest that the vasculature is continually expanding while in a state of re-established quiescence in enlarging MRL-lpr/lpr lymph nodes.

### Treatment with VEGFR inhibitor SU5416 reduces anti-dsDNA titers cells without long term effects on vascular expansion

**Figure 2:**
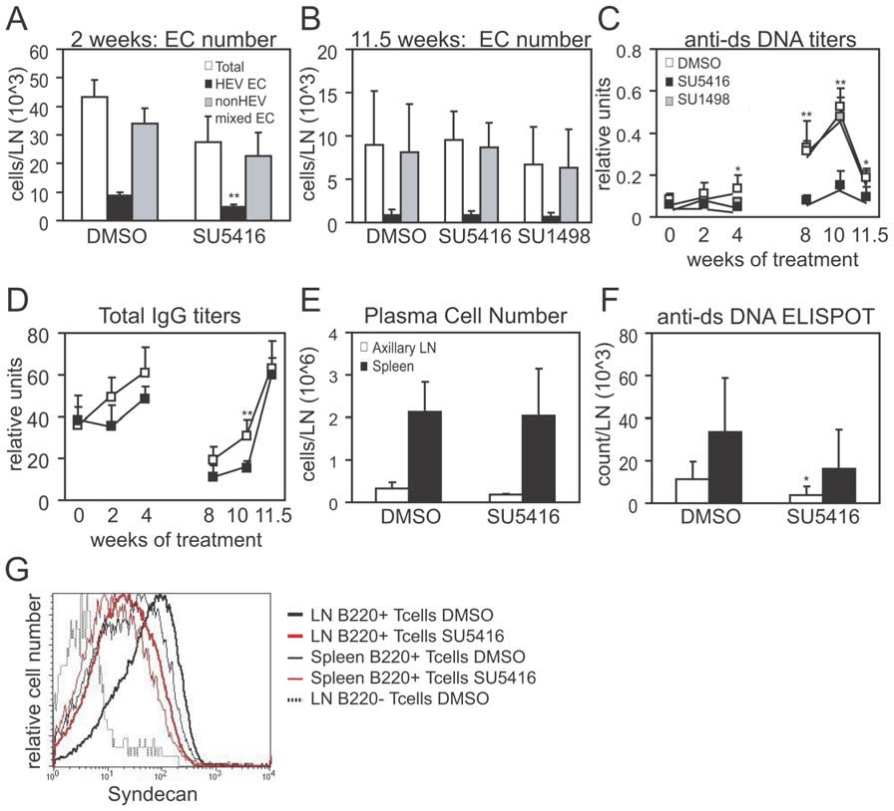
SU5416 reduces anti-dsDNA and alters T cell phenotype but without sustained effect on vascular growth. (A–D) MRL-lpr/lpr mice (JAX 006825) were treated for either 2 weeks (A) or 11.5 weeks (B–D) with indicated treatments starting at 8 weeks of age. (E–G) MRL-lpr/lpr (JAX 000485) were treated for 5–6 weeks with SU5416 starting at 10 weeks of age. (A) Endothelial cell numbers in brachial lymph nodes after 2 weeks of SU5416 treatment. n = 3 mice per condition. (B) Endothelial cell numbers in brachial lymph nodes after 11.5 weeks of SU5416 treatment. n = 4 DMSO mice and 3 SU5416 mice. Representative of 2 similar experiments. (C) Anti-dsDNA titers and (D) total IgG titers in serum over time. For (C–D), ELISA for serum from 0, 2, and 4 weeks was run independently from serum at 8, 10, and 11.5 weeks. n = 4 mice per treatment except at 11.5 weeks, when n = 3 for SU5416 (see text). Representative of 2 similar experiments. (E) Number of plasma cells per organ. n = 4 mice per treatment over 3 experiments. (F) Number of anti-dsDNA-secreting cells as determined by ELISPOT. n = 4 mice per treatment over 3 experiments. (G) Syndecan levels on indicated T cell populations from SU5416 or DMSO-treated mice. Representative of 4 mice over 3 experiments. For (A–F), * = p<.05 and * = p<.01 when SU5416 treatment compared to DMSO treatment using t-test.

We wanted to ask whether inhibiting lymph node vascular expansion could reduce the ensuing autoantibody generation. We treated mice with SU5416, a small molecule receptor tyrosine kinase inhibitor that inhibits VEGF receptors as well as having activity against other receptor tyrosine kinases such as Flt-3 and PDGFR-beta [42]–[46]. Treatment of 2 month-old MRL-lpr/lpr mice for 2 weeks, when endothelial cell numbers and anti-dsDNA titers were rising (Fig. 1B, 1C), resulted in reduced numbers of HEV endothelial cells (Fig. 2A), suggesting that SU5416 had the potential capacity to reduce vascular expansion, at least in the short term.

We then treated mice with SU5416 for 11.5 weeks, starting at 2 months of age. To ask if any potential effects could be attributable to the VEGF receptor-inhibiting activity of SU5416, we treated other mice with SU1498, another inhibitor of VEGF receptor tyrosine kinases that does not inhibit Flt-3 [44], [47]. One mouse out of 4 treated with SU5416 for each of 2 experiments died at 10–11 weeks of treatment. Urine protein of these mice was not any higher than other mice in the group (data not shown) and these deaths were potentially consistent with toxicity of SU5416 that has been previously described [48]. Endothelial cells numbers were unchanged by either SU5416 or SU1498 in brachial lymph nodes (Fig. 2B), suggesting that compensatory mechanisms may have overcome the prolonged VEGF receptor blockade or that VEGF is not an important mediator of the prolonged vascular expansion in MRL-lpr/lpr nodes. CD11c^hi^ presumed dendritic cells were also not consistently reduced, suggesting potential compensation for any SU5416-mediated Flt3 blockade (data not shown). Total lymph node cellularity was also not consistently altered by SU5416 (data not shown). Remarkably, however, SU5416 attenuated the rise in anti-dsDNA titers (Fig. 2C) with less effect on total serum IgG (Fig. 2D).

In order to understand whether the reduced anti-dsDNA in serum could reflect at least in part fewer anti-dsDNA-expressing plasma cells in lymph nodes, we examined a separate cohort of MRL-lpr/lpr mice that was treated with SU5416 for 5–6 weeks starting at 3 months of age. We enumerated plasma cells by flow cytometry, gating on B220^lo^syndecan^pos^CD3^neg^ cells. Total plasma cell numbers were unchanged with SU5416 treatment in axillary nodes and spleen (Fig. 2E), but anti-dsDNA-secreting cells as detected by ELISPOT assay were reduced in number in lymph nodes (Fig. 2F). Together, these results suggested that SU5416 treatment reduced the number of anti-dsDNA-secreting plasma cells with a lesser effect on disrupting the general plasma cell population.

The double negative T cells in MRL-lpr/lpr mice account for a large portion of the cellularity in MRL-lpr/lpr lymph nodes over time [36] (Fig. 1H) and may help promote autoantibody responses [49]. The number of double negative T cells in lymph nodes was not consistently affected by SU5416 (data not shown). These cells express syndecan [37], and remarkably, syndecan levels on these cells were downregulated in lymph nodes upon SU5416 treatment (Fig. 2G). In comparison, double negative cells in spleen expressed lower and more heterogenous levels of syndecan than in lymph nodes (Fig. 2G), suggesting lymphoid tissue-specific regulation of double negative T cell phenotype. Splenic double negative T cells showed modest reduction of syndecan with SU5416 (Fig. 2G). Although the function of syndecan on the double negative T cells is unknown, the phenotypic change in the double negative T cells suggested the possibility of a functional change with SU5416.

### Double negative T cells are located in ectopic follicles that lack normal T zone stromal and other elements and SU5416 normalizes lymphocyte-microenvironment relationship

**Figure 3:**
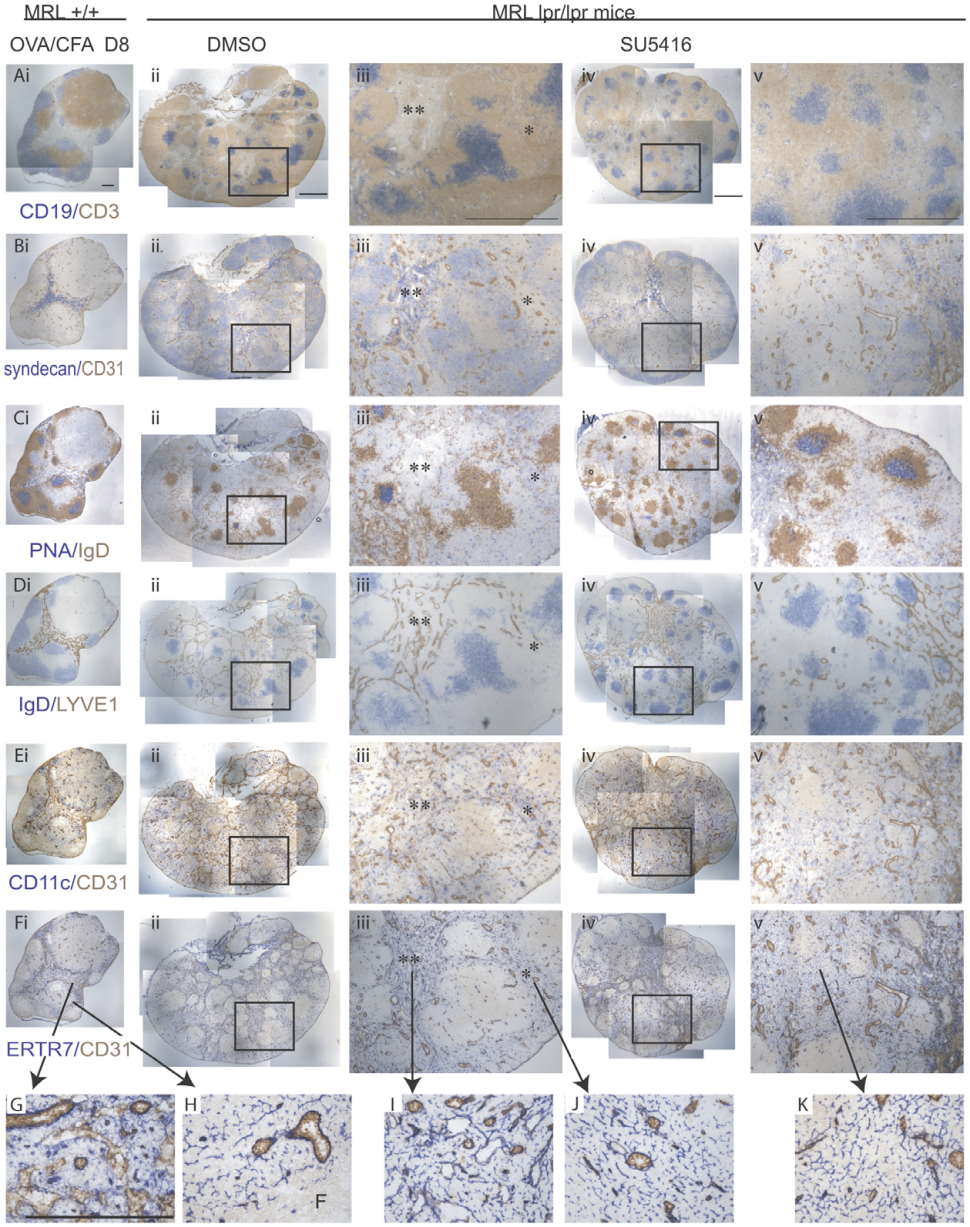
Follicles of double negative T cells exclude T zone constituents and SU5416 normalizes the microenvironment. MRL-lpr-lpr mice were treated with SU5416 or DMSO vehicle for 11.5 weeks starting at 8 weeks of age and lymph nodes were examined. For comparison to a stimulated wild-type lymph node, 10 week old MRL+/+ mice were immunized with OVA/CFA and draining brachial lymph nodes were examined at day 8. (Part i of A–F), nearby sections from a single day 8 MRL+/+ brachial lymph node were stained as indicated. (Part ii of A–F), nearby sections from a single brachial lymph node from a DMSO-treated MRL-lpr/lpr mouse were stained as indicated. (Part iii of A–F), higher magnification of boxed area in corresponding images in part ii. (Part iv of A–F), nearby sections from a single brachial lymph node from a SU5416 -treated MRL-lpr/lpr mouse were stained as indicated. (Part v of A–F), higher magnification of boxed area in corresponding images in part iv. (G–H) Higher magnification of (G) medullary and (H) T zone areas in (Fi). “F” denotes follicle. (I–J) Higher magnification of (I) medullary-like and (J) T zone-like areas in (Fiii). (K) Higher magnification of normalized T zone area in (Fv). Scale bars represent 500 um.

We stained sections to ask whether anatomic alterations accompanied the reduction in anti-dsDNA and double negative T cell syndecan levels. A brachial lymph node from a 10 week old MRL-+/+ mouse at day 8 after immunization with ovalbumin in CFA (OVA/CFA) is shown for comparison purposes. In contrast to the distinct B cell follicles and T zones in the stimulated MRL+/+ node (Fig. 3Ai) and consistent with the observations of Lieberum and Hartmann [8], MRL-lpr/lpr nodes were filled with well-delineated ectopic follicles consisting of a central B cell zone and a corona of T cells (Fig. 3Aii, 3Aiii). Consistent with the localization of double negative T cells in the corona of the follicles [8], the coronae were syndecan^med^ (Fig. 3Bii, 3Biii). Some of these follicles contained PNA+ germinal centers within the central B zone (Fig. 3Cii, 3Ciii). Some of the follicles were surrounded by “bare” CD19^neg^CD3^neg^ areas (Fig. 3Aii, 3Aiii double asterisks) containing syndecan^hi^ plasma cells (Fig. 3Bii, 3Biii double asterisks) and LYVE1+ lymphatic sinuses (Fig. 3Dii, 3Diii double asterisks). The accumulation of plasma cells and lymphatic sinuses are hallmarks of medullary cords (Fig. 3Bi and 3Di) and the delineation of the ectopic follicles by plasma cell- and lymphatic-rich areas suggests that many of the ectopic follicles are located within a greatly engorged medulla.

Some of the follicles had a cortical location (3Aii, 3Aiii), similar to that of normal B cell follicles (Fig. 3Ai), although these follicles also had the abnormal corona of syndecan^med^ (double negative) T cells (Fig. 3Bii, 3Biii). Furthermore, while cortical B cell follicles are normally juxtaposed to (Fig. 3Ai) and can receive input from the subcapsular sinus [50], [51], a thick layer of syndecan+ T cells appeared interposed between the cortical follicles and the subcapsular sinus in the MRL-lpr/lpr nodes (Fig. 3Aii, 3Aiii, 3Bii, 3Biii).

Syndecan^neg^ T cells that normally inhabit the paracortex could be observed in patches between some of the ectopic follicles (Fig. 3Aiii, 3Biii, asterisk). CD11c^pos^ dendritic cells are normally concentrated in the T zone (Fig. 3Ei). In the MRL-lpr/lpr nodes, they were relatively sparse in the zones occupied by syndecan^med^CD3^pos^ T cells within the follicles, and appeared to more densely populate the areas occupied by normal syndecan^neg^CD3^bright^ T cells (Fig. 3Eii, 3Eiii, asterisks). Similarly, the large, thick-walled, CD31^hi^ HEVs are mainly localized in the T zone and medulla in wild-type mice (Fig. 3Bi, 3Ei, 3G, 3H), but tended to be relatively sparse within the double negative T cell-rich corona of the ectopic follicles (Fig. 3Bii, 3Biii, 3Eii, 3Eiii, 3Fii, 3Fiii) as compared to the medullary (double asterisk region in Fig. 3Biii, 3Eiii and 3Fiii)and T zone-like areas surrounding the follicles (single asterisk region in Fig. 3Biii, 3Eiii and 3Fiii). The MRL-lpr/lpr lymph nodes, then, had the appearance of medullary engorgement and were occupied mostly by ectopic follicles full of double negative T cells and B cells. Normal paracortical T cells and associated T zone elements appeared to be squeezed into small areas between some of the ectopic follicles.

B cell patches remained distributed throughout the lymph node after treatment with SU4516 (Fig. 3Aiv–v), but the medulla appeared more centralized and less engorged, as indicated by the more centrally located syndecan^hi^ plasma cells and LYVE1 staining (Fig. 3Biv, 3Div). The well-circumscribed ectopic follicles filled with syndecan^med^CD3^pos^ cells appeared smaller and fewer and were mostly localized near or within the normalized medulla (Fig. 3Aiv, 3Biv, 3Div). Consistent with the flow cytometry findings (Fig. 2 G), CD3^pos^ T cells in the paracortex had appreciably reduced levels of syndecan after SU5416 (Fig. 3Biv-2Bv), giving a more normalized appearance to the paracortex. Additionally, more even distribution of CD11c^pos^ cells and HEVs (Fig. 3Eiv–3Ev) contributed to the impression of a normalized paracortex. The syndecan^med^CD3 ^pos^ cells that were detectable remained localized to the outer boundaries of the cortical follicles after SU5416 (Fig. 3Aiv, 3Biv–v). SU1498, in contrast, did not have these effects on the lymph node anatomical organization (data not shown). Together these results suggest that there is disruption of ectopic follicles and normalization of the lymph node organization in association with the reduction in anti-dsDNA and double negative T cell phenotype changes after SU5416.

We examined the distribution of ER-TR7, a component of the extracellular matrix secreted by FRCs [27], to better understand the alterations in the lymph node architecture in the MRL-lpr/lpr mice with and without SU5416 treatment. ER-TR7 staining is normally quite bright and relatively disorganized in the medulla (Fig. 3Fi, 3G) while it shows a distinct reticular pattern in the T zone and (Fig. 3Fi, 3H). It is normally largely excluded from the B cell follicles, defining the border between the T zone and the B cell follicles [24]–[27] (see Fig. 3H). In the MRL-lpr/lpr nodes, bright medullary-type ER-TR7 staining could be observed in the medullary cord-like areas (Fig. 3Fiii double asterisks and Fig. 3I). In the areas occupied by syndecan^neg^CD3^hi^ T cells (Fig. 3Aiii asterisks, 3Biii asterisks), normal T zone reticular ER-TR7 staining was seen (Fig. 3Fiii asterisks, 3J). Remarkably, despite the T cells within the ectopic follicles (Fig. 3Aii–iii, 4B), only sparse ERTR7 staining was seen within the ectopic follicles (Fig. 3Fii–iii, 4E). ER-TR7, then, appeared excluded from the zone of double negative T cells within the ectopic follicles in MRL-lpr/lpr mice.

With SU5416 treatment, a more normal central pattern of bright medullary ER-TR7 staining (Fig. 3Fiv) was consistent with the idea of a normalized medullary compartment. In the normalized paracortical T zone, areas occupied by T cells now no longer lacked ER-TR7 (Fig. 3Aiv–v, 3Fiv–v, 4C, 4F). The pattern of ER-TR7 in these T cell areas was reticular, resembling that of a normal T zone (Fig. 3Fiv–v, 3K). Notably, ER-TR7 remained sparse in area just under the subcapsular sinus occupied by the remaining syndecan^med^CD3^pos^ T cells (Fig. 3Biv, 3Fiv). These results together indicate that T zone microenvironmental constituents including ER-TR7, HEVs, and dendritic cells are relatively lacking in the T cell-rich areas of the ectopic follicles and that SU5416-induced disruption of ectopic follicles is accompanied by normalized association between T cells and T zone microenvironmental constituents. These results suggest a potential scenario whereby double negative T cells and the associated B cells within the ectopic follicles are sequestered and protected from a normal T zone microenvironment.

**Figure 4:**
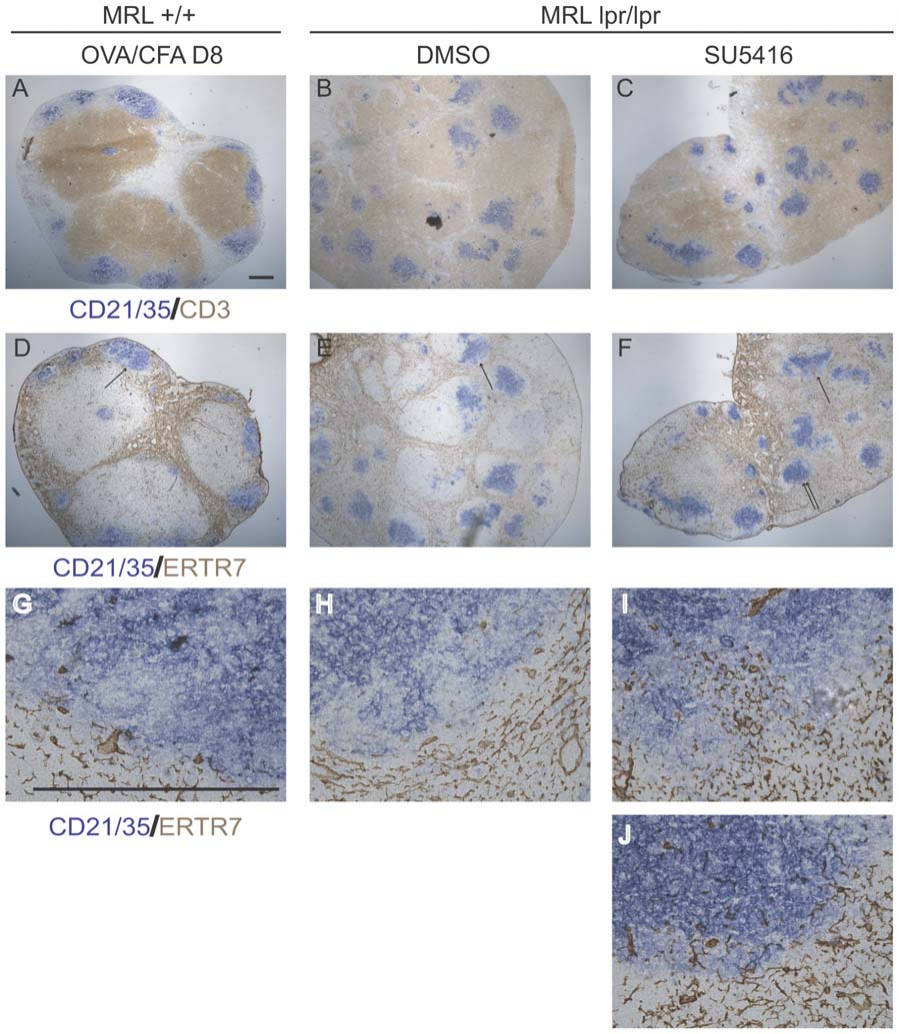
SU5416 induces infiltration of ER-TR7 into the B cell areas within ectopic follicles. MRL-lpr-lpr mice were treated with SU5416 or DMSO vehicle for 11.5 weeks starting at 8 weeks of age and lymph nodes were examined. For comparison to a stimulated wild-type lymph node, 10 week old MRL+/+ mice were immunized with OVA/CFA and draining brachial lymph nodes were examined at day 8. (A, D) nearby sections from a single brachial lymph node from a DMSO-treated MRL-lpr/lpr mouse were stained as indicated. (B, E) Nearby sections from a single axillary lymph node from a DMSO-treated MRL-lpr/lpr mouse were stained as indicated. (C,F) Nearby sections from a single axillary lymph node from a SU5416-treated MRL-lpr/lpr mouse were stained as indicated. (G) Higher magnification of B cell follicle border indicated in (D, arrow). (H) Higher magnification of ectopic follicle border indicated in (E, arrow). (I, J) Higher magnification of border of B cell areas indicated in (F). (I) is magnification of area denoted by single arrow and (J) is magnification of area denoted by double arrows. Scale bars represent 500 um.

The relative lack of ER-TR7 staining within the ectopic follicles is reminiscent of the ER-TR7 exclusion from normal B cell follicles. In support of a B cell follicle-type microenvironment within the ectopic follicles, these follicles contained a network of CD21/CD35^hi^ cells (Fig. 4B, 4E, 4H) resembling follicular dendritic cells (FDCs), the specialized stromal cells of the B cell follicles (Fig. 4A, 4D, 4G). Upon SU5416 treatment, the patches containing CD21/CD35^hi^ cells (FDCs) along with CD21/CD35^med^ cells (B cells) could still be observed (Fig. 4C, 4F). However, while ER-TR7 was excluded from the CD21/CD35+ zones even in instances where ER-TR7 was directly abutting the CD21/CD35+ zones in control MRL-lpr/lpr mice (Fig. 4H), SU5416 treatment led to the appearance of reticular ER-TR7 staining within the CD21/CD35+ patches. The ER-TR7 staining within the CD21/CD35+ zone appeared continguous with the ER-TR7 staining at the border of the zones, giving the appearance of an invasive process (Fig. 4I, 4J). Interestingly, Cyster and colleagues recently showed that ablation of FDCs resulted in invasion of ER-TR7 and other T zone constituents into the areas formerly occupied by B cell follicles [26], suggesting the possibility that SU5416 could have disrupted ectopic follicles by initially affecting FDC integrity. Together, our results suggested that the ectopic follicles containing double negative T cells and B cells in MRL-lpr/lpr mice are in a microenvironment that at least in part resembles that of B cell follicles, and that disruption of the ectopic follicles exposes both T and B cells of the follicles to a T zone-type microenvironment.

## Discussion

Our results showed that lymph node hypertrophy in the MRL-lpr/lpr model of lupus is accompanied by vascular expansion and also revealed that double negative T cells and B cells are organized within ectopic follicles that were disrupted in association with SU5416-induced attenuation of the anti-dsDNA response. The T cell-rich corona of the ectopic follicles lacked a normal T zone microenvironment, and SU5416-induced follicular disruption normalized the T cell-microenvironment association. The association of the microenvironmental alterations with anti-dsDNA responses and double negative T cell phenotype alterations raises the possibility that these phenomena are related and that microenviromental alterations might contribute to regulating autoimmune responses. T zone FRCs have been shown to have a regulatory capacity [18]–[23], suggesting the possible scenario that sequestration of lymphocytes in ectopic follicles maintains autoimmune responses at least in part by protecting them from regulation by T zone FRCs. Such a scenario would also suggest that targeting the relationship between the lymphocytes and the microenvironment may be a means by which to inhibit autoimmune responses.

Our results showed that the characteristics of vascular expansion accompanying the progressive lymph node enlargement in MRL-lpr/lpr mice are similar to the vascular expansion that occurs after model immunizations. By examining at long intervals in the MRL-lpr/lpr mice, we were likely to have missed any proliferative burst that characterizes the first days after acute immunization of wild-type mice [29], [30]. However, the modestly elevated endothelial cell proliferation rate, the reduced HEV trafficking efficiency, and the moderately elevated VEGF all were consistent with the idea that the blood vessels were in the state of re-established quiescence that follows the initial burst of proliferative expansion [31]. The increased ratio of CD11c^hi^ cell:CD11c^med^ cell further supports this idea and also raises the possibility that, similar to lymph nodes after model immunization, CD11c^hi^ cells may also regulate this phase of quiescence in MRL-lpr/lpr nodes. Short term VEGF receptor blockade with SU5416 reduced HEV numbers, suggesting that, similar to lymph nodes after model immunization [30], [40], the elevated VEGF levels contributed to the vascular expansion. The ineffectiveness of long term blockade, though disappointing in not allowing us to understand the importance of vascular expansion to the autoimmune response, was similar to the transient effectiveness of VEGF blockade that we had previously observed for homeostatic HEV proliferation [40]. The similarity in vascular alterations and potential regulatory mechanisms suggest that the vasculature undergoes a somewhat stereotypical response upon lymph node stimulation and that mechanisms gleaned from studying vascular regulation after model immunization can be useful for understanding lymph node vascular regulation in spontaneous chronic autoimmune models. It will be interesting in the future to understand whether there are characteristics of the vasculature or regulatory mechanisms that are specific for promoting or limiting autoimmune responses.

Our results suggest the possibility that localization of double negative T cells with B cells in ectopic follicles is related to the maintenance of the anti-dsDNA response. Whether and how they are related remains to be fully elucidated, but double negative T cells from lupus patients can help B cells to generate an anti-dsDNA response [52]–[54], and these cells in MRL-lpr/lpr mice express IL-17 and can contribute to pathogenic humoral responses [49]. These features in addition to their compartmentalization with B cells raise the interesting possibility that they resemble a type of follicular T helper cell that can potentially contribute to pathology by helping to promote anti-dsDNA and other autoantibody responses. SU5416 induced the reduction of syndecan on the double negative cells, and it would be interesting to understand whether the level of syndecan is related to their ability to generate IL-17 and/or contribute to humoral responses. While we do not currently understand whether lymphocyte function or the regulation of the ectopic follicle compartmentalization was more directly affected by SU5416, the SU5416 treatment resulted in exposure of the double negative T cells and associated B cells to a more T zone-like microenvironment that included ER-TR7 and a greater complement of HEVs and dendritic cells. Recent studies have shown that T zone FRCs can play a regulatory role in limiting T cell activation and proliferation by a variety of mechanisms [18]–[23]. Thus, an attractive potential scenario is that the disruption of ectopic follicles contributed to reduced anti-dsDNA at least in part by exposing the double negative T cells and accompanying B cells within the follicles to the regulatory influence of T zone FRCs.

The targets of the SU5416 that are involved in the reduction in anti-dsDNA responses and in lymph node alterations are currently unclear. The lack of effect of SU1498 on these parameters would suggest that VEGF receptor inhibition is not involved. SU5416 also inhibits Flt3, which is important for dendritic cell development [44], [45], [55]. CD11c^hi^ presumed dendritic cells were not reduced in number with long term SU5416 treatment, but whether SU5416-mediated blockade affected dendritic cell function was not assessed. Dendritic cells are important for the generation of autoantibodies in MRL-lpr/lpr mice [56] and we and others have shown that CD11c+ dendritic and other cells can regulate FRC proliferation and function, and potentially in a direct manner [21], [29], [31]; it is possible that SU5416 induced alterations in dendritic cell function that normalized the stromal compartment and reduced anti-dsDNA responses. Similarly, blockade of receptor tyrosine kinases such as CSF-1R, c-kit, or c-ret by SU5416 [43], [57], [58]could also have affected other cells involved in regulating the stromal elements and the anti-dsDNA response. SU5416 also has activity against PDGFR-beta [59], which is expressed by FRCs [60], and could potentially modulate their function. Treatment with imatinib, an inhibitor of PDGF receptor and c-kit receptor kinases, reduced lymph node size and ameliorated nephritis in MRL-lpr/lpr mice [61], and it would be interesting to understand whether imatinib has the same effects as SU5416 on anti-dsDNA responses and lymph node organization.

Identifying the critical SU5416 targets that are important for the effects in the MRL-lpr/lpr lymph nodes may have implications for potential new therapies in lupus. SU5416 was tested in clinical trials for its anti-angiogenic and anti-tumor properties and was discontinued as a clinical agent because of side effects and suboptimal pharmacokinetics. Sunitinib is a subsequent generation VEGF receptor inhibitor that has a similar profile of activity against multiple receptor tyrosine kinases and that is orally available. It is in clinical use as a first line agent in patients with metastatic clear cell renal cell carcinoma [59], [62], suggesting the possibility of relatively quick translation from pre-clinical results into clinical practice for lupus and other systemic autoimmune diseases. However, anti-VEGF activity may not be desirable in lupus, as the renal vasculature is dependent on podocyte-expressed VEGF [63] and NZB/WF1 mice treated with antibodies to VEGFR2 had accelerated nephropathy [64]. SU5416 induced only slightly more proteinuria in the MRL-lpr/lpr mice that we examined at 11.5 weeks of treatment (30+ to 100+ in SU5416-treated mice as compared to <30 to 30+ in DMSO-treated mice; data not shown), but more specific agents that do not compromise renal function would seem to be more desirable.

Our results provide new insight into the features of the immune system that are associated with autoantibody generation. Although more work will be needed to fully establish the relationship between autoantibody generation and lymph node microenvironmental alterations, these associations raise the possibility that physical sequestration of autoimmune cells from regulatory microenvironmental elements could potentially be a mechanism for sustaining pathogenic responses and that targeting the association of lymphocytes with the microenvironment may be a strategy by which to control autoimmune responses. Testing this model and understanding the generalizability of our findings in other autoimmune models will be helpful to better understand the potential utility of this strategy and also to provide further insight into mechanisms of disease.
